# A multifactorial analysis of FAP to regulate gastrointestinal cancers progression

**DOI:** 10.3389/fimmu.2023.1183440

**Published:** 2023-05-30

**Authors:** Jialing Cai, Depeng Yang, Handi Sun, Lixing Xiao, Fang Han, Mengmeng Zhang, Lu Zhou, Meiyi Jiang, Qinghua Jiang, Yu Li, Huan Nie

**Affiliations:** School of Life Science and Technology, Harbin Institute of Technology, Harbin, Heilongjiang, China

**Keywords:** FAP, fibroblast, gastrointestinal cancers, immunology, macrophage polarization

## Abstract

**Background:**

Fibroblast activation protein (FAP) is a cell-surface serine protease that has both dipeptidyl peptidase as well as endopeptidase activities and could cleave substrates at post-proline bond. Previous findings showed that FAP was hard to be detected in normal tissues but significantly up-regulated in remodeling sites like fibrosis, atherosclerosis, arthritis and embryonic tissues. Though increasing evidence has demonstrated the importance of FAP in cancer progression, no multifactorial analysis has been developed to investigate its function in gastrointestinal cancers until now.

**Methods:**

By comprehensive use of datasets from The Cancer Genome Atlas (TCGA), Clinical Proteomic Tumor Analysis Consortium (CPTAC), scTIME Portal and Human Protein Atlas (HPA), we evaluated the carcinogenesis potential of FAP in gastrointestinal cancers, analyzing the correlation between FAP and poor outcomes, immunology in liver, colon, pancreas as well as stomach cancers. Then liver cancer was selected as example to experimentally validate the pro-tumor and immune regulative role of FAP in gastrointestinal cancers.

**Results:**

FAP was abundantly expressed in gastrointestinal cancers, such as LIHC, COAD, PAAD and STAD. Functional analysis indicated that the highly-expressed FAP in these cancers could affect extracellular matrix organization process and interacted with genes like COL1A1, COL1A2, COL3A1 and POSTN. In addition, it was also observed that FAP was positively correlated to M2 macrophages infiltration across these cancers. To verify these findings *in vitro*, we used LIHC as example and over-expressed FAP in human hepatic stellate LX2 cells, a main cell type that produce FAP in tumor tissues, and then investigate its role on LIHC cells as well as macrophages. Results showed that the medium from FAP-over-expressed LX2 cells could significantly promote the motility of MHCC97H and SK-Hep1 LIHC cells, increase the invasion of THP-1 macrophages and induce them into pro-tumor M2 phenotype.

**Conclusion:**

In summary, we employed bioinformatic tools and experiments to perform a comprehensive analysis about FAP. Up-regulation of FAP in gastrointestinal cancers was primarily expressed in fibroblasts and contributes to tumor cells motility, macrophages infiltration and M2 polarization, revealing the multifactorial role of FAP in gastrointestinal cancers progression.

## Introduction

Gastrointestinal cancers refer to tumors in esophagus, stomach, colon, liver as well as pancreas, currently regarded as one of the most leading cause of cancer death and the major obstruction in cancer treatment (1, 2). Numerous evidences have demonstrated the key role of immune microenvironment in the occurrence and development of gastrointestinal cancers (3), however, the regulatory mechanisms need further exploration. With continuous development and improvement of bioinformatic tools, it is possible to reveal the significance and correlation of specific genes in immune microenvironment regulation, providing an opportunity to evaluate the potential of these genes as novel prognosis markers and treatment target across gastrointestinal cancers (4).

Fibroblast activation protein (FAP) is a constitutively active serine peptidase with both dipeptidyl peptidase and collagenase activity (5). Previous findings revealed that FAP was rare to be detected in healthy tissues, notably, FAP had a high expression trend in some kinds of malignant tumors, such as breast cancer, colorectal cancer and pancreatic cancer (6–10). High expression of FAP in these cancers was reported to estimate worse outcomes in patients and involved in tumor progression via diverse mechanisms. For instance, FAP was found able to promote migration and invasion of cancer cells by binding to ENO1 and activating NF-κB signaling pathway in colorectal adenocarcinoma (COAD) (11). In stomach adenocarcinoma (STAD), high FAP expression in tumor tissues is always accompanied by increased micro-vessel density (12), while after FAP knock-out or pharmacological inhibition, tumor growth and microvascular density could be decreased (13), suggesting that FAP was involved in angiogenesis as well. The effect of FAP in tumor immune system was not investigated until recent years. In a mouse model of pancreatic adenocarcinoma (PAAD), Feig C and colleagues found that the depletion of FAP positive cells contributed to improved anti-CTLA-4 or anti-PD-L1 immunotherapy efficacy, revealing the immune suppressive effect of FAP in cancers (14). Similar findings were also observed in mouse model of COAD and STAD, results showed that co-injection of cancer cells and FAP positive cells led to anti-PD-1 treatment resistance in mice (15, 16). Though these findings suggested a significant role of FAP in gastrointestinal cancers progression, a multifactorial and comprehensive analysis is still needed.

Our current study utilized bioinformatic methods to give a description on the functions of FAP across gastrointestinal cancers and then verified these findings by *in vitro* experiments. Results showed that FAP was up-regulated in gastrointestinal cancers and involved in tumor cell mobility, macrophages infiltration as well as M2 polarization process. The study not only revealed the multifactorial role of FAP in gastrointestinal cancer progression but also provided the first evidence that M2 macrophages played dominant roles underlying FAP immune-suppressive effects, revealing a novel target for future treatment options.

## Materials and methods

### Gene expression analysis

Data collected from the Cancer Genome Atlas (TCGA) were used to visualize the mRNA expression level of FAP in various human cancers and their normal counterparts. Furthermore, the TISBID website was used to examine the expression of FAP mRNA and the grading of tumors. The relationship between FAP mRNA expression in the normal, tumor and metastasis site was evaluated using TNMplot online server.

### Protein expression and immunohistochemistry staining

In order to investigate the expression of FAP protein in different human tumors and their normal counterparts, the UALCAN program was used. UALCAN developed protein expression analysis using data collected from the Clinical Proteomic Tumor Analysis Consortium (CPTAC). The IHC staining images of FAP in different tumor tissues and normal tissues were obtained from HPA (Human Protein Atlas) dataset.

For IHC staining of LIHC tissues from patients, antigens were retrieved by sodium citrate for 10 min. Later the sections were incubated with 5% BSA at room temperature for 1 h to get rid of unspecific bindings. Primary antibodies were diluted with PBS and an overnight incubation was conducted at 4 °C. Following primary antibodies, the sections were washed and incubated by secondary antibodies for 1 h the next day. Afterwards, color reaction was carried out using DAB kit. All images were captured using an optical microscope.

### Survival prognosis and ROC diagnosis analysis

Sangerbox webserver was utilized to evaluate the survival outcomes of FAP in different human cancers. To explore the diagnosis value of FAP in various cancers, the pROC R package was used for statistical analysis and ggplot2 R package was used to create the receiver operating characteristic (ROC) curve. ROC curves of FAP with Area Under the Curve (AUC) more than 0.7 was regarded as high diagnostic values in different types of human cancers.

### FAP methylation analysis

DNA methylation is a kind of DNA chemical modification and behaves as an essential regulator of gene transcription. FAP DNA methylation analysis using data collected from TCGA database was conducted using UALCAN. Analysis of the correlation between FAP expression and gene promoter methylation was developed for each type of cancer.

### Protein-protein interaction and functional enrichment analysis

FAP co-expression data was downloaded from TCGA dataset. |log_2_FC| > 1.5, adj *p* < 0.05 was used as a standard to obtain FAP-correlating genes. These genes were enriched by Gene Ontology (GO) [including biological processes (BP), cellular components (CC), and molecular function (MF)] and KEGG pathway analyses. Then the STRING website was used to acquire top 20 FAP-interacting genes. Vein diagram was used to conduct analysis to compare FAP-correlating and interacting genes in different human cancers. The “Gene_Corr” module of Timer2.0 was used to generate heatmap or correlation curve of FAP-correlating and interacting genes, which contains partial correlation and *p* value.

### Immune reactivity analysis

Estimation of Immune Cells in Malignant Tumor Tissues Using Expression Data (ESTIMATE) is a method to investigate the degree of stroma or immune cell infiltration into tumors according to existing gene expression data. The ESTIMATE was used to estimate immune scores for each tumor. The correlation between FAP expression and immune cell infiltration was calculated using MCPcounter by Sangerbox webserver. In addition, the relationship between FAP and immune check-point, tumor mutation burden (TMB) and microsatellite instability (MSI) was also evaluated using SangerBox webserver.

### Single-cell sequencing analysis

The expression of FAP, MRC1 and NOS2 in different cellular component of tumor was obtained and analyzed using scTIME Portal online server. The species and cancer type were first tabbed to select a dataset. Then the gene name was inserted to visualize FAP, MRC1 and NOS2 expression in different cells in malignant tumor. The interactions among fibroblasts, tumor cells and macrophages were also obtained and analyzed using CellphoneDB analysis via scTIME Portal online server.

### Cell lines and cell culture

The human hepatic stellate LX2 cell line, liver cancer MHCC97H and SK-Hep1 cell line, monocyte THP-1 cell line was purchased from Stem Cell Bank, Chinese Academy of Sciences. All cells were cultured at 5% CO_2_ and 37°C in DMEM or RPMI-1640 medium and routinely examined to exclude mycoplasma contamination by Genetic Testing Biotechnology Corporation (Suzhou, China).

### Cell transfection and cell stimulation

FAP plasmid and its corresponding control plasmid were designed and constructed. During transfection, lipofectamine 3000 reagent was used according to the manufacturer’s protocol. The culture medium was collected 48 h after transfection and then filtered to remove cellular debris. The culture medium was then transferred to MHCC97H and SK-Hep1 liver cancer cells as well as PMA-treated THP-1 cells. The migration and invasion of cancer cells were detected using wound healing assay and transwell assay. The polarization state of THP-1 cells was detected by qRT-PCR.

### Wound healing and transwell assay

For wound healing assay, the MHCC97H and SK-Hep1 cells were planted in 6-well plates at a density of 4×10^^5^, scratches were made in the middle of the well. The cells were treated with conditioned medium collected from LX2 cells for 48 h, then the wound closure was measured.

For transwell assay, 1.5×10^^4^ cells were treated with serum-free medium and inoculated in the upper chamber. The LX2 cells were planted in the lower chamber and treated with complete medium. After 24 h culture, cells that migrated across the membrane were stained using 1% crystal violet and photographed.

### Quantitative reverse-transcriptase PCR analysis and western blotting

Total RNA was extracted from LX2 and THP-1 cells using TRIzol reagent and 1 μg total RNA was then reverse-transcribed. Quantitative reverse-transcriptase PCR was conducted using a reaction mix of SYBR Green and the relative expression of target genes was compared using ΔΔCt method and GAPDH served as the endogenous gene.

Total protein was lysed from cells by RIPA lysis buffer added with protease inhibitor. Protein concentration was measured using the BCA Protein Assay Kit. SDS-PAGE was used to separate the proteins and PVDF membrane was used to transfer the proteins. 5% skim milk was used to block unspecific bindings at room temperature for 1 h and then primary antibodies were used to incubate the membrane. After overnight incubation, the membrane was washed and incubated with secondary antibodies. Finally, the protein bands were visualized by chemiluminescence system.

### Statistical analysis

The experimental data were presented as mean ± S.E.M. and analyzed using Graphpad Prism 7.0 software. The difference between two groups were analyzed using Student’s t test. *p* < 0.05 was considered as significant.

## Results

### FAP is abnormally up-regulated and correlated to poor prognosis in gastrointestinal cancers

**Figure 1:**
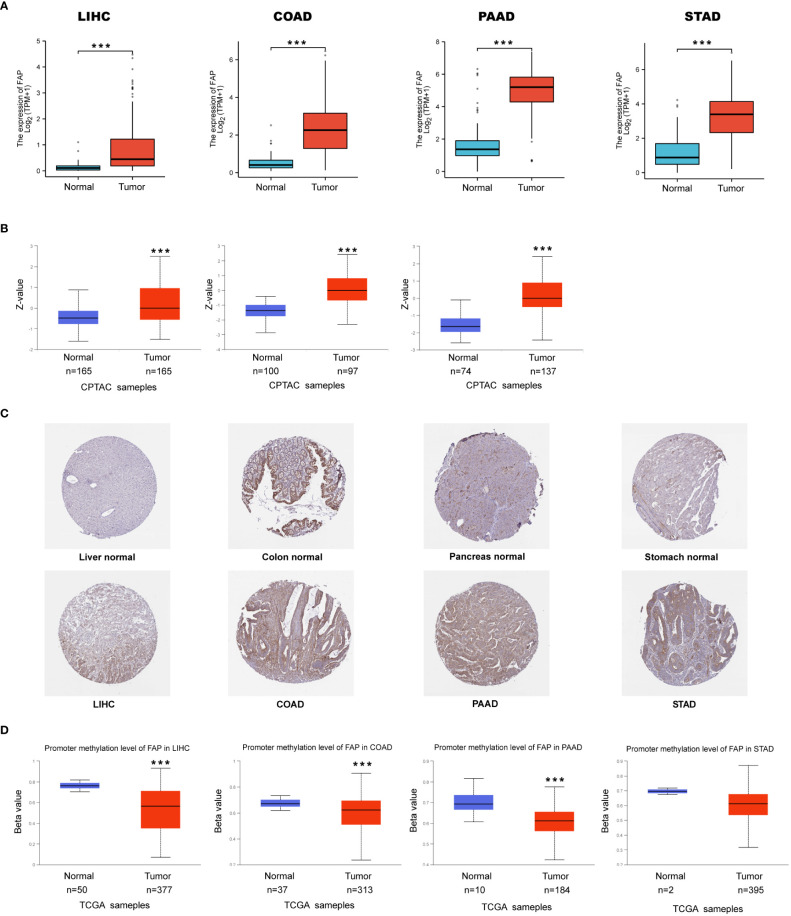
FAP up-regulation by DNA methylation in gastrointestinal cancers. **(A)** Expression of FAP mRNA in gastrointestinal cancers. **(B, C)** Expression of FAP protein in gastrointestinal cancers versus normal tissues (left side), and IHC staining for normal tissue (middle) and tumors (right side) from HPA database. **(D)** Differential expression of FAP promotor methylation in gastrointestinal tumors versus normal tissues. ^***^ *p* < 0.001.

First, TCGA dataset was used to examine the expression of FAP mRNA in tumors and adjacent normal tissues. According to our findings, FAP mRNA was increased in most of the tumors (22/31) (**Supplementary Figure 1A**). Notably, we noticed that FAP mRNA was commonly increased in gastrointestinal cancers such as LIHC, COAD, PAAD and STAD, which attracted our attention (**Figure 1A**). In this regard, we further analyzed the expression of FAP at protein level in these gastrointestinal tumors using the National Cancer Institute’s CPTAC dataset. Results indicated that the expression of FAP protein was significantly up-regulated in LIHC, COAD, PAAD as compared to their normal counterparts, which was also validated by IHC staining pictures acquired from the Human Protein Atlas (HPA) dataset. The protein expression data of FAP in STAD was not found using CPTAC, higher FAP protein expression was still observed in the IHC staining pictures (**Figures 1B, C**). Then we intended to investigate whether the up-regulation of FAP in tumor tissues correlated to DNA methylation of FAP promotor using UALCAN online tool. Beta value ranging from 0 (unmethylated) to 1 (fully methylated) in **Figure 1D** indicates the level of DNA methylation, 0.5 to 0.7 indicates hypermethylated, while hypomethylated when the value ranging from 0.25 to 0.3. Our results suggested that the methylation of FAP promotor was significantly lower in LIHC, COAD and PAAD as compared to normal tissues. Though the data showed that DNA methylation at FAP promotor in STAD is unaffected, we speculated that this may be due to limited case numbers since there is still a downregulated tendency (**Figure 1D**).

Then TNM plot online server was used to compare expression of FAP mRNA in normal, tumor and metastasis sites. Results indicated that FAP mRNA was significantly up-regulated in tumors of liver, colon, pancreas as compared to normal tissues. Furthermore, this pattern would maintain between the metastatic and cancerous sites in colon (**Supplementary Figure 1B**). Subsequently, we tried to figure out the association between FAP mRNA and cancer stages. By TISDIB online web server, we found that FAP was positively correlated to the disease stage of COAD as well as PAAD (**Supplementary Figure 1C**). Moreover, FAP could affect the survival probability of gastrointestinal cancer patients and has high diagnostic accuracy in the model of ROC analysis for the cancers (**Supplementary Figures 2**, **3**).

### FAP is correlated to extracellular matrix organization in gastrointestinal cancers

**Figure 2:**
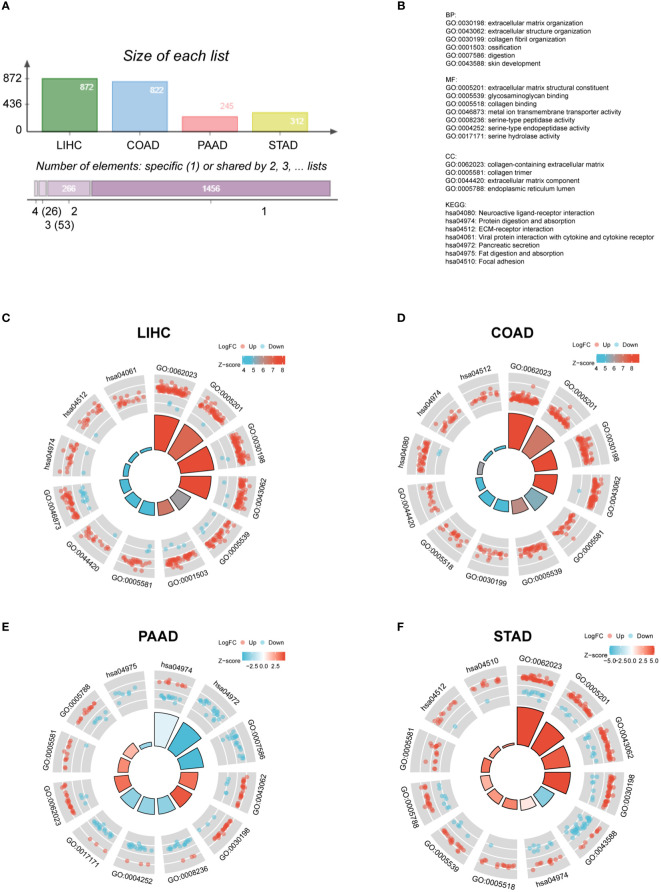
GO and KEGG analysis of FAP in different gastrointestinal cancers. **(A)** FAP-correlating genes in different gastrointestinal cancers. **(B)** GO and KEGG functional annotations. GO and KEGG analysis of FAP in **(C)** LIHC. **(D)** COAD. **(E)** PAAD. **(F)** STAD.

Afterwards, it is essential to investigate the functions of FAP across different gastrointestinal cancers. FAP-correlating proteins with |log_2_FC| > 1.5, adj *p* < 0.05 were obtained from TCGA datasets. Totally 872, 822, 245 and 312 genes were identified correlated to FAP in LIHC, COAD, PAAD and STAD, respectively (**Figure 2A**). To further investigate the functional significance of FAP in these cancers, FAP-correlating proteins obtained from different cancers were reanalyzed using Gene Ontology (GO) enrichment analysis. Results showed that FAP may be closely associated with extracellular matrix or structure organization process across all these four tumors (**Figures 2B–F**). Undoubtedly, the data also suggested that FAP was involved in specific functions of certain tumor, for instance, FAP is also associated with digestion function in PAAD (**Figure 2E**).

**Figure 3:**
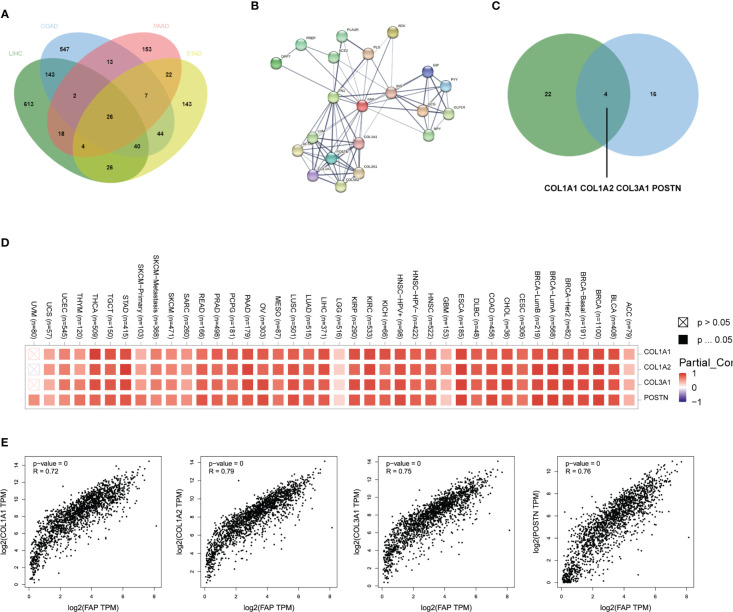
FAP-associated protein network interactions in different gastrointestinal cancers. **(A)** FAP-correlating proteins in different gastrointestinal cancers. **(B)** A map of top 20 FAP-interacting proteins analyzed by STRING database. **(C)** Vein diagram showing overlap between FAP-correlating and interacting proteins in different human cancers. **(D)** Heatmap showing both FAP-correlating and interacting proteins in tumor tissues. **(E)** Expression correlation analysis between FAP and FAP-correlating and interacting proteins in tumor tissues.

Then vein diagram identified totally 26 genes that were commonly correlated to FAP expression across these four tumors (**Figure 3A**). At the meanwhile, top 20 FAP-interacting genes were extracted from STRING database and displayed as a protein-protein interaction network (**Figure 3B**). After comparing proteins from these two lists, 4 genes including COL1A1, COL1A2, COL3A1 and POSTN were identified both correlated and interacted with FAP in gastrointestinal cancers (**Figure 3C**). A heatmap created by Timer2.0 then validated significant positive correlation between these four genes and FAP (**Figure 3D**). Besides, we also used Timer2.0 to obtain correlation analysis plots of all these 4 genes with FAP (**Figure 3E**): COL1A1 (R = 0.72), COL1A2 (R = 0.79), COL3A1 (R = 0.75) and POSTN (R = 0.76).

### FAP is correlated to M2 macrophage infiltration in gastrointestinal cancers

**Figure 4:**
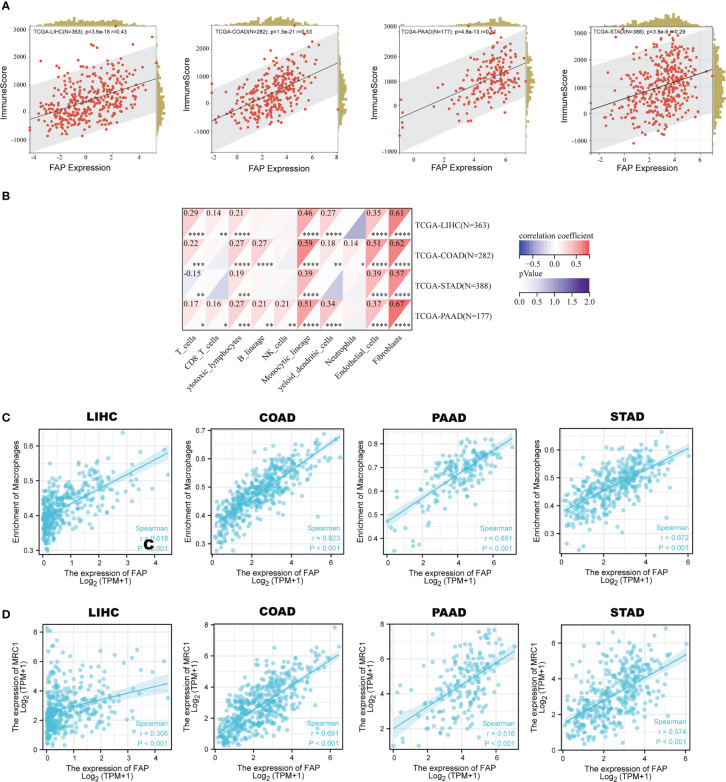
Correlation between FAP and M2 macrophages infiltration in gastrointestinal cancers. **(A)** Correlation between FAP expression and immune scores across gastrointestinal cancers. **(B)** Analysis of immune cell infiltration correlated to FAP expression across gastrointestinal cancers. **(C)** Scatter plots showing the correlation between FAP expression and macrophage infiltration in gastrointestinal cancers. **(D)** Scatter plots showing the correlation between FAP expression and M2 macrophage marker MRC1 in gastrointestinal cancers.

Since extracellular matrix organization process plays crucial roles in building the immune-suppressive tumor microenvironment (TME) (17), then we tried to figure out whether FAP was involved in the immune-regulatory process. We utilized the ESTIMATE algorithm to calculate the correlation between FAP expression and immune scores. Results showed that FAP was positively correlated to immune scores in LIHC, COAD, PAAD and STAD (**Figure 4A**). In addition, significant correlation between FAP and immune check-points, MSI and TMB also suggested that FAP was involved in cancer immunology (**Supplementary Figures 4A–C**). We then intended to examine the relationship between FAP and the infiltration of different immune cells using MCPcounter. Results indicated that FAP was significantly correlated to monocyte across all the four gastrointestinal cancers (**Figure 4B**). Monocytes are the main source of macrophages and FAP was found positively correlated to the infiltration of macrophages across all these cancers (**Figure 4C**), suggesting that FAP may be involved in cancer immunology by regulating macrophages functions.

As is known, macrophages are a kind of immune cells that could exert opposite effects depending on their polarization phenotypes, with M1 suppressive while M2 promotive on tumor progression. The M1 macrophages usually expressed markers like NOS2 (iNOS), TNF, IL1B, while M2 expressed MRC1 (CD206), Arg-1, IL-10 and so on. By single-cell sequencing analysis, we found that MRC1 was highly and primarily expressed in macrophages across these four gastrointestinal cancers (**Supplementary Figures 5A–D**). Gene correlation analysis indicated a positive correlation between the expression of FAP and MRC1 in the gastrointestinal cancers (**Figure 4D**), suggesting that FAP was possibly involved in M2 macrophage infiltration in gastrointestinal cancers.

### FAP is primarily expressed in fibroblasts in gastrointestinal cancers

**Figure 5:**
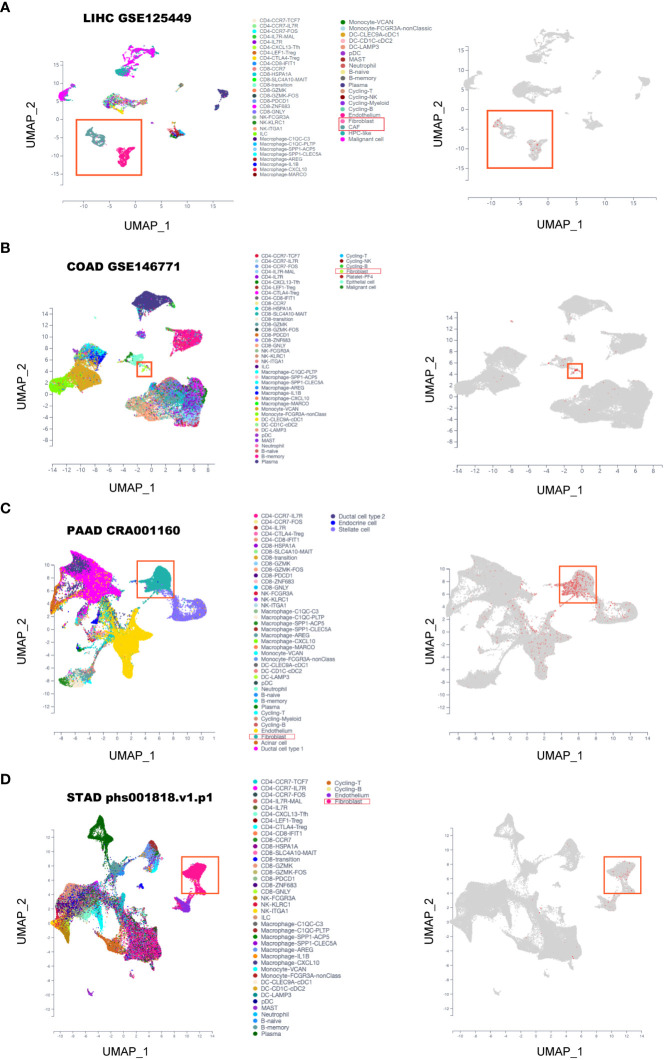
Single-cell sequencing analysis of FAP expression in gastrointestinal cancers. **(A)** LIHC. **(B)** COAD. **(C)** PAAD. **(D)** STAD.

It has been reported that the four FAP-correlating and interacting genes were mainly expressed in fibroblasts of tumors (18, 19), then we intended to investigate whether FAP was also expressed in fibroblasts in gastrointestinal cancers. By using scTIME Portal online server for single-cell sequencing analysis. We found that FAP was exclusively expressed in fibroblasts as well as cancer associated fibroblasts (CAFs) in LIHC (GSE125449) (**Figure 5A**). Similarly, same conclusions were obtained from single-cell sequencing analysis of COAD (GSE146771), PAAD (cra001160) as well as STAD (phs001818.v1.p1) (**Figures 5B–D**). By further analysis using CellphoneDB analysis, we found there are strong interactions between fibroblasts and tumor cells as well as macrophages in gastrointestinal cancer tissues (**Supplementary Figures 6A–D**). These results indicated that FAP was primarily expressed in fibroblasts and its role on tumor progression was achieved via affecting the interaction between fibroblasts, tumor cells and macrophages.

### FAP-over-expressed fibroblasts promoted cancer cell motility in LIHC

**Figure 6:**
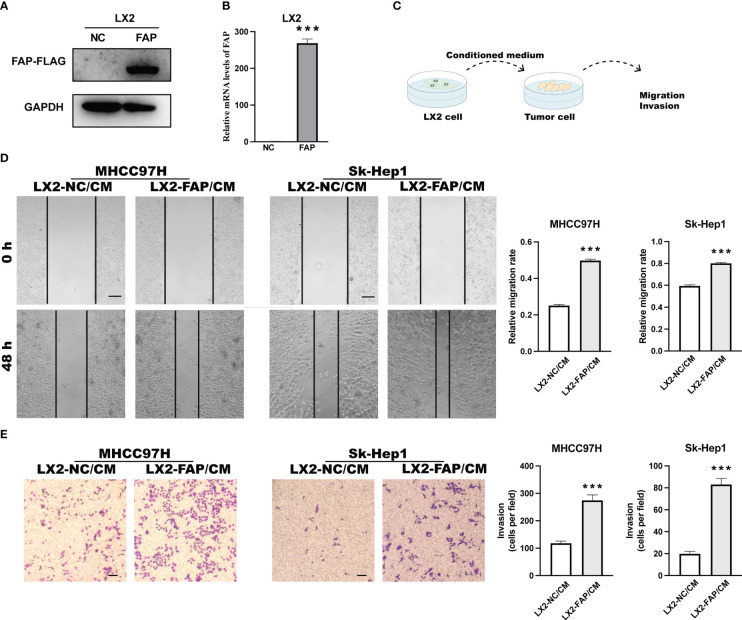
Experimental validation of relationship between FAP expression and tumor cell motility in LIHC. **(A, B)** FAP expression after FAP overexpression in human hepatic stellate LX2 cell line. **(C)** Experimental diagram. **(D)** Wound healing assay investigating the effect of FAP on tumor cell migration. **(E)** Transwell assay investigating the effect of FAP on tumor cell invasion. ^***^ *p* < 0.001. Scale bar = 200 μm.

First, we investigated the role of FAP in fibroblasts on tumor cells *in vitro*. LIHC is one of the gastrointestinal cancers that influenced by FAP, we overexpressed FAP in human hepatic stellate LX2 cell line, the key source of fibroblast in LIHC and then collected the cell medium 48 h later (**Figures 6A, B**). The medium was used to treat liver cancer MHCC97H and SK-Hep1 cells for 48 h or 24 h, tumor cell migration and invasion was examined using wound healing test as well as the transwell invasion assay (**Figure 6C**). Results showed that the conditioned medium from FAP-over-expressed LX2 cells could significantly promote the cell invasion and migration rate of both MHCC97H and Sk-Hep1 cells as compared to NC group (**Figures 6D, E**), suggesting that FAP in fibroblast is involved in tumor cell motility process in LIHC.

### FAP-over-expressed fibroblasts promoted macrophages infiltration and M2 polarization in LIHC

**Figure 7:**
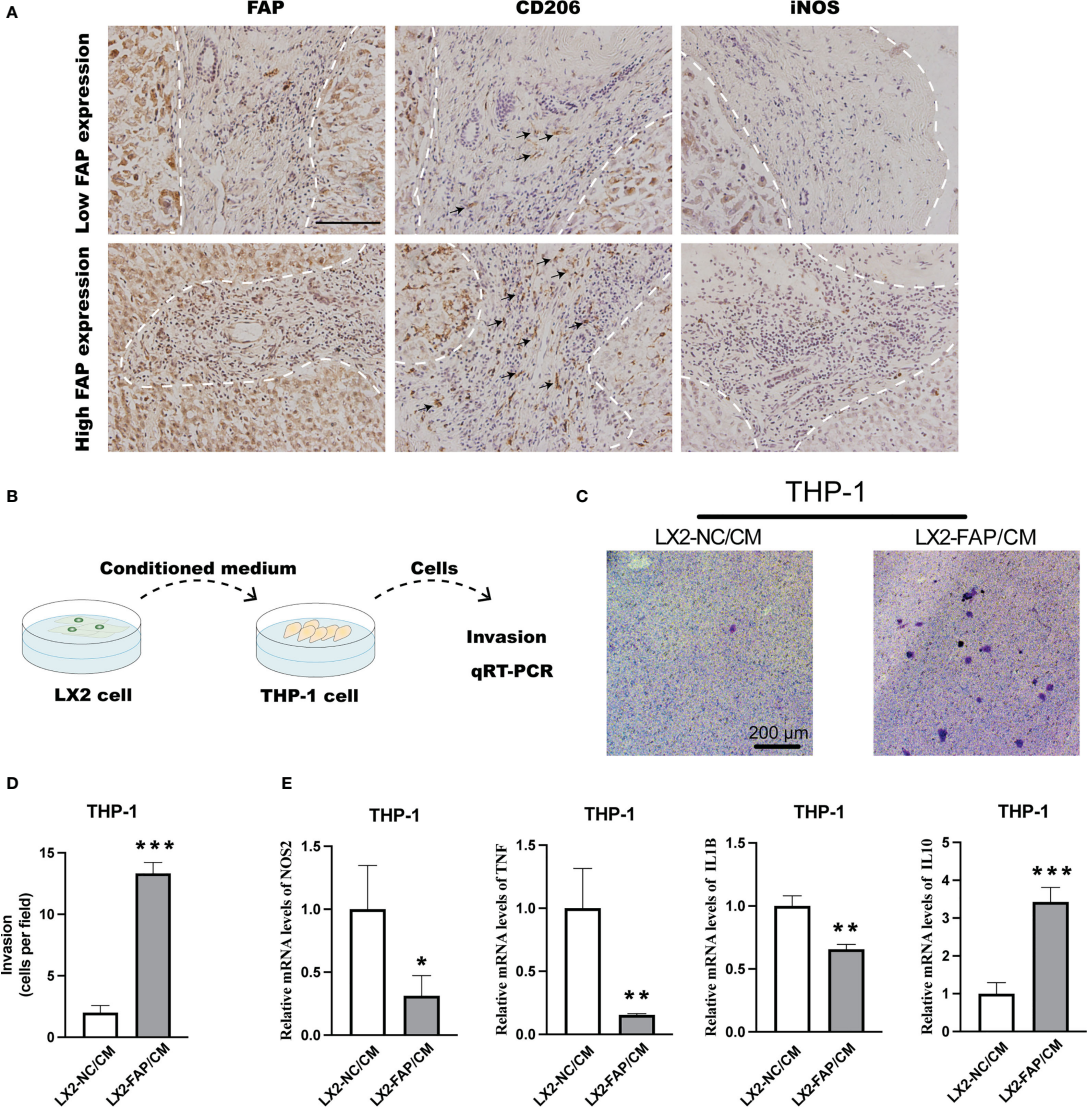
Experimental validation of relationship between FAP expression and M2 macrophage polarization. **(A)** IHC staining of FAP, CD206 and iNOS in LIHC tissues. **(B)** Experimental diagram. **(C, D)** Transwell assay investigating the effect of FAP on macrophage invasion. **(E)** Transwell co-culturing system investigating the effect of FAP on M2 macrophage polarization. ^*^ *p* < 0.05, ^**^ *p* < 0.01, ^***^ *p* < 0.001. Scale bar = 1 mm.

In LIHC, IHC staining suggested that more M2 macrophages were presented in tumor tissues of patients with high FAP expression (**Figure 7A**). In *in vitro* experiments, THP-1 monocyte was first treated by PMA for 48 h and then stimulated by the conditioned medium collected from LX2 cells (**Figure 7B**). The conditioned medium collected from FAP-over-expressed LX2 cells could significantly promote the invasion rate of THP-1 macrophages as compared to the NC group (**Figures 7C, D**). Then qRT-PCR was conducted to examine M1 or M2 macrophage marker alterations in THP-1 cells. Results showed that conditioned medium from FAP-over-expressed LX2 cells could decrease the expression of M1 markers like iNOS, TNF-α and IL-1β but increase the expression of M2 markers like IL-10 in macrophages as compared to NC group, transforming the macrophages into M2 pro-tumor phenotype (**Figure 7E**). These results indicated that FAP in fibroblasts is involved in macrophages infiltration and M2 polarization in LIHC

In summary, FAP is primarily expressed in fibroblasts of gastrointestinal cancers and promoted cancer progression via promoting tumor cell motility as well as macrophages infiltration and M2 polarization.

## Discussion

FAP is a serine peptidase that share 70% of the sequence identity with the enzyme dipeptidyl-peptidase to facilitate extracellular matrix reorganization and promote tumor malignancies. The current study started with analyzing the mRNA level of FAP in a list of human cancers, where it was found that FAP mRNA was significantly up-regulated in 22 of 31 cancers. Interestingly, we found that FAP mRNA was consistently up-regulated in gastrointestinal cancers including LIHC, COAD, PAAD as well as STAD. Similar findings were observed at protein level via CPTAC and HPA dataset and the expression of FAP positively correlated with poor outcomes of gastrointestinal cancers. As gastrointestinal cancers are increasingly prevalent in the world and possibly account for 20% of cancer cases (20), we suspected that FAP could be a potential gastrointestinal cancer biomarker and instructive for future treatment options.

Previous evidence has already demonstrated a significant role of FAP in tumor progression through multiple mechanisms. For instance, the invasion and migration ability of cancer cells was significantly promoted after coculturing with FAP positive CAFs isolated from the stroma tissue of breast cancer patients (21). Inhibition of FAP via anti-FAP IgG1 antibody or knock-down by siRNA could reverse FAP-mediated migration and invasion promotion (22). Besides, FAP was also involved in angiogenesis of TME, depletion of FAP and dipeptidyl peptidase 4 (DPP4) in CAFs led to decease of vascularization in colorectal cancer, while such effect was not observed when DPP4 was depleted alone (23). In the present study, we found that FAP was generally involved in extracellular matrix organization process across all these four gastrointestinal cancers by analyzing FAP-correlated genes in LIHC, COAD, PAAD and STAD. After comparing the commonly correlated genes of FAP across gastrointestinal cancers with top 20 FAP-interacting genes extracted from STRING database, 4 genes including COL1A1, COL1A2, COL3A1 and POSTN were identified both correlated and interacted with FAP. It is noteworthy that all these 4 genes have been demonstrated to be involved in extracellular matrix organization process (18, 19), validating our previous findings.

Since extracellular matrix contributes to the construction of the immune microenvironment surrounding tumor cells (17, 24, 25), we found that FAP was positively correlated to immune scores across different gastrointestinal cancers by the ESTIMATE algorithm, suggesting that FAP is involved in cancer immune regulation. More importantly, we found that FAP was significantly correlated to macrophages infiltration across gastrointestinal cancers, suggesting that the immune-regulatory effect of FAP in gastrointestinal cancers is possibly mediated by macrophages. It is commonly accepted that macrophages can be classified into two phenotypes, the classically activated pro-inflammatory M1 phenotype as well as the alternatively activated anti-inflammatory M2 phenotype (26). In most cases, M1 phenotype of macrophages exhibited positive correlation to better prognosis and longer survival times of patients with cancer like lung cancer (27), colon cancer (28), breast cancer (29) and so on, while the M2 phenotype exhibited opposite roles. It is recorded that the majority of macrophages surrounding the tumor exhibited an M2 phenotype and can assist the tumor cells in proliferation, metastasis, angiogenesis, immune escape and drug resistance (30–32). By bioinformatic analysis, we found that FAP was positively correlated to the expression of M2 macrophages marker MRC1 across gastrointestinal cancers, suggesting that FAP may be involved in regulating M2 macrophages in these cancers.

Afterwards, we intended to validate the findings of FAP on macrophages by experiments. FAP was found primarily expressed in fibroblasts across these cancers by single-cell sequencing analysis, verified previous findings that FAP behaves as a marker of CAFs (33). Furthermore, strong interactions have been identified between fibroblasts and macrophages, suggesting that fibroblasts may be involved in regulating macrophages functions across gastrointestinal cancers. For instance, the fibroblasts-derived CSF-1, IL-6 and CCL2 has been found to promote macrophages infiltration and M2 phenotype polarization process in pancreas cancer (34). As for the effect of FAP in fibroblasts on macrophages functions, though FAP in fibroblasts have been demonstrated to be closely located with pro-tumor macrophages in tumor tissues and involved in macrophages migration process (12, 35), the role of FAP on macrophages functions has not been further investigated yet. In the present study, we over-expressed FAP in human hepatic stellate LX2 cells for 48 h and collected medium to treat macrophages. The results showed that medium from FAP-over-expressed LX2 cells could promote the invasion ability of macrophages and increase their transformation into M2-like phenotype, providing the first evidence that FAP is involved in macrophage M2 polarization in gastrointestinal cancers. Furthermore, we also investigated the effect of FAP in fibroblast on tumor cells. Results showed that medium from FAP-overexpressed LX2 cells could promote tumor cell migration as well as invasion process. Based on these results, we found that the up-regulation of FAP could promote gastrointestinal cancers progression through promoting tumor cell motility as well as macrophages infiltration and M2 polarization.

Although our study is not the first work to demonstrate the significant role of FAP in tumor, there is still some innovations. First, we are the first to give a comprehensive illustration of FAP across gastrointestinal tumors using bioinformatics methods and then validate significant findings using experimental methods. Second, our study laid the foundation for detailed studies of the correlation between FAP expression and diverse immune cell infiltrations, first revealing the role of FAP on inducing M2 macrophages polarization to promote tumor progression across gastrointestinal cancers. Regretful, these results were obtained only focusing on FAP that expressed in fibroblasts, though previous evidence has demonstrated that fibroblasts contribute to main source of FAP in tumor tissues, FAP could also be detected in other kinds of cells like tumor cells, endothelial cells, monocytes, lymphocytes at lower concentration (36, 37), in this regard, their effects in tumor progression cannot be neglected and required more experiments for analysis.

In conclusion, the present study provided the first multifactorial analysis of FAP in gastrointestinal cancers, revealing that the up-regulation of FAP in these cancers is correlated to tumor progression through promoting tumor cell motility as well as macrophages infiltration and M2 polarization. These findings may provide more evidence for FAP as gastrointestinal cancers treatment targets.

## Data availability statement

The original contributions presented in the study are included in the article/**Supplementary Material**. Further inquiries can be directed to the corresponding authors.

## Author contributions

JC, DY and HS contributed equally to this work. JC and DY contributed conception and design of the study. HS and LX organized the database. FH, MZ, LZ and MJ performed the statistical analysis. JC, DY and HS wrote the first draft of the manuscript. LX, FH, MZ, LZ and MJ wrote the sections of the manuscript. All authors contributed to manuscript revision, read and approved the submitted version.

## Supplementary material

Supplementary Figure 1 Correlation between FAP expression and clinical outcome of gastrointestinal cancers. **(A)** Expression of FAP mRNA in pan-cancers. **(B)** Expression of FAP in normal, tumor and metastatic sites of gastrointestinal cancers. **(C)** Expression of FAP across different stages of gastrointestinal cancers. ^**^ *p* < 0.01, ^***^ *p* < 0.001

Supplementary Figure 2 Cox regression analysis of FAP in pan-cancers. **(A)** LIHC. **(B)** COAD. **(C)** PAAD. **(D)** STAD.

Supplementary Figure 3 ROC curves indicating the AUC of FAP in gastrointestinal cancers. **(A)** LIHC. **(B)** COAD. **(C)** PAAD. **(D)** STAD.

Supplementary Figure 4 Correlation between FAP and immune check-points, MSI and TMB. **(A)** immune check-points. **(B)** MSI. **(C)** TMB.

Supplementary Figure 5 Single-cell sequencing analysis of NOS2 and MRC1 in gastrointestinal cancers. **(E)** LIHC. **(F)** COAD. **(G)** PAAD. **(H)** STAD.

Supplementary Figure 6 Single-cell sequencing analysis of fibroblasts and macrophages interactions in gastrointestinal cancers. **(A)** LIHC. **(B)** COAD. **(C)** PAAD. **(D)** STAD.
