# Supplementary material for: A multifactorial analysis of FAP to regulate gastrointestinal cancers progression
Source: Front Immunol. 2023 May 30;14:1183440. doi: 10.3389/fimmu.2023.1183440 (PMC10262038; doi:10.3389/fimmu.2023.1183440)
Supplement: Supplementary Figure 1 — Correlation between FAP expression and clinical outcome of gastrointestinal cancers. (A) Expression of FAP mRNA in pan-cancers. (B) Expression of FAP in normal, tumor and metastatic sites of gastrointestinal cancers. (C) Expression of FAP across different stages of gastrointestinal cancers. ** p < 0.01, *** p < 0.001 [file Presentation_1.pdf]

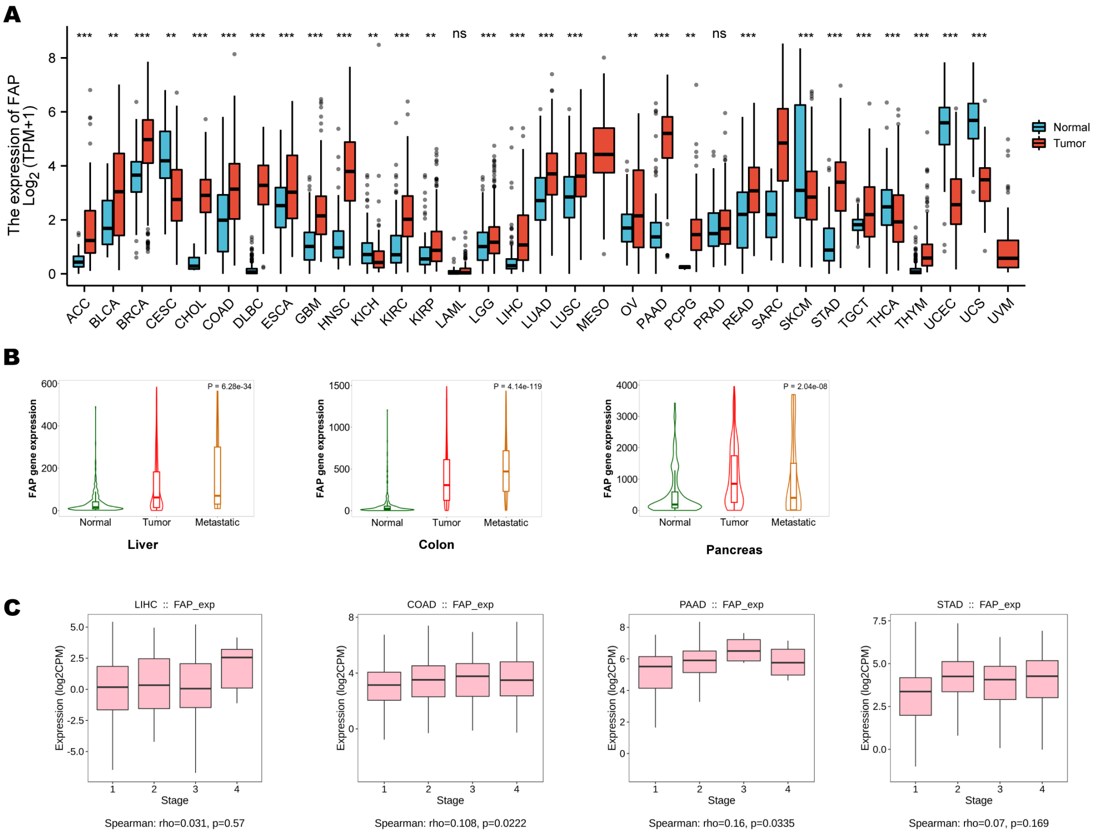

Supplementary Fig. 1 Correlation between FAP expression and clinical outcome of gastrointestinal cancers. (A) Expression of FAP mRNA in pan-cancers. (B) Expression of FAP in normal, tumor and metastatic sites of gastrointestinal cancers. (C) Expression of FAP across different stages of gastrointestinal cancers. **p < 0.01, ***p < 0.001

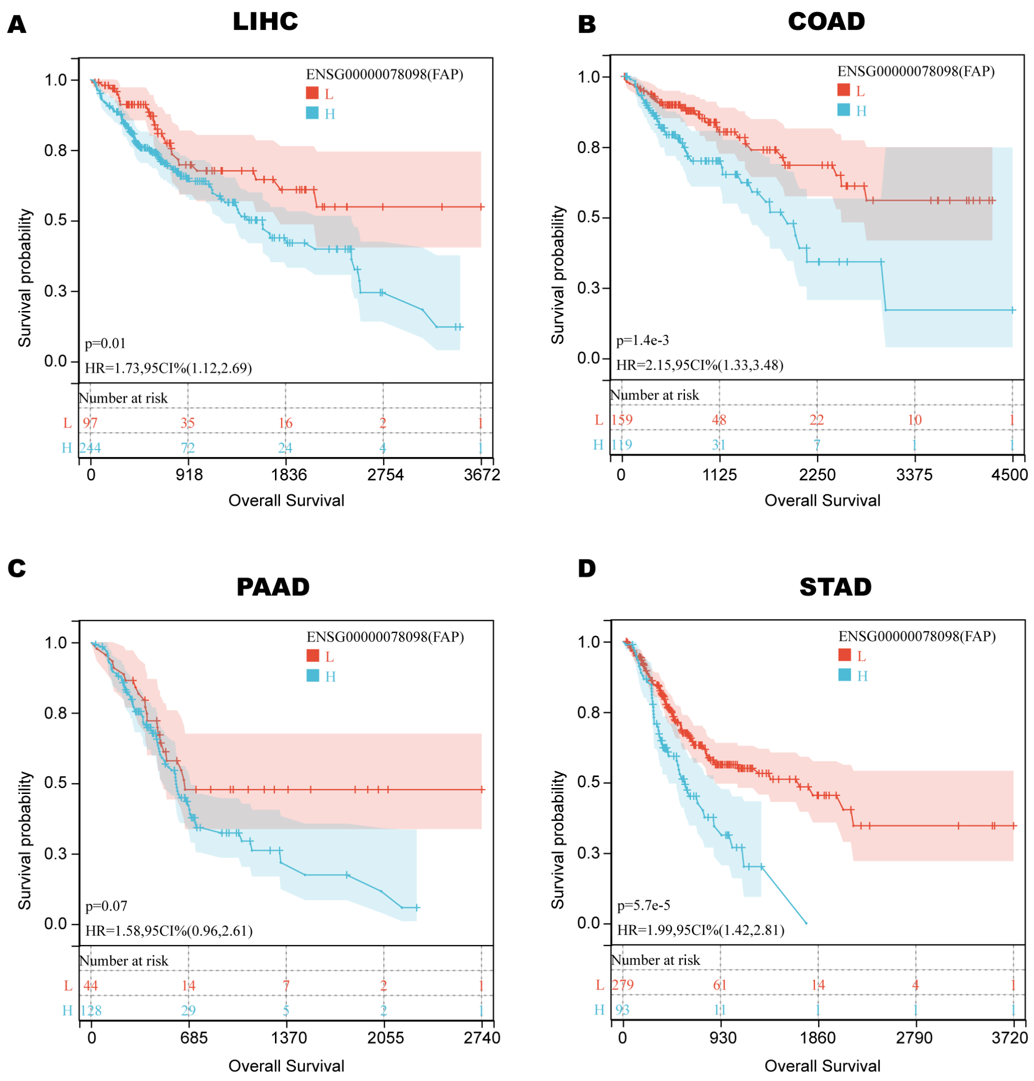

Supplementary Fig. 2 Cox regression analysis of FAP in pan-cancers. (A) LIHC. (B) COAD. (C) PAAD. (D) STAD.

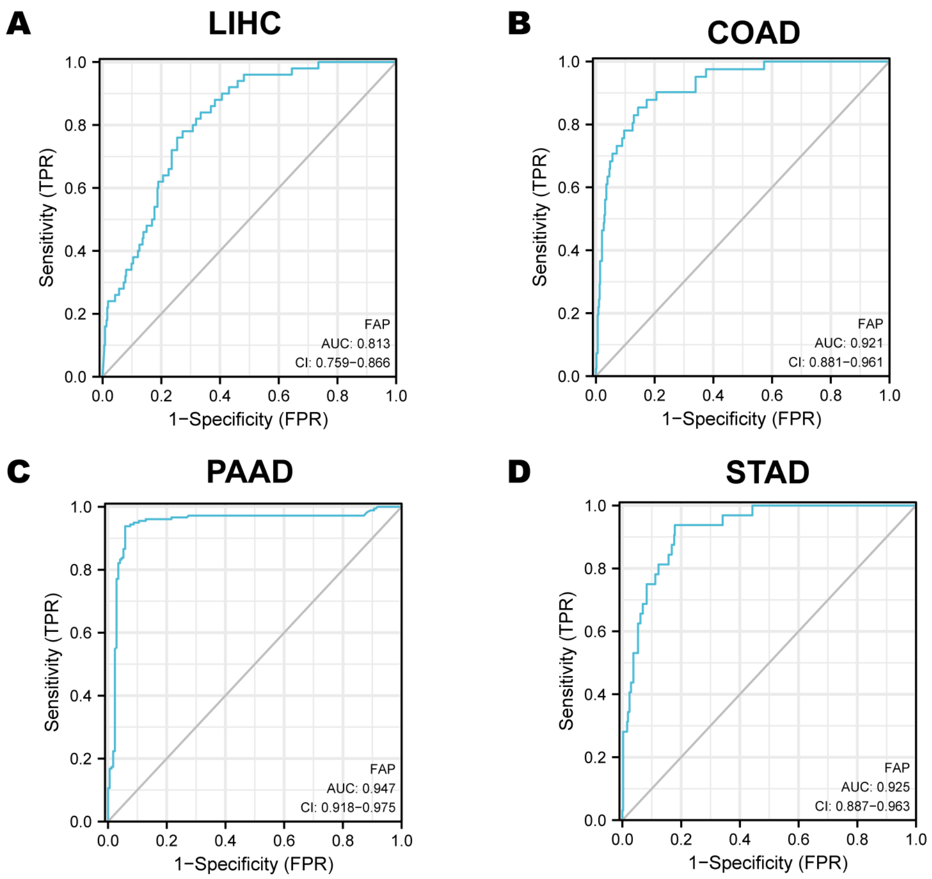

Supplementary Fig. 3 ROC curves indicating the AUC of FAP in gastrointestinal cancers. (A) LIHC. (B) COAD. (C) PAAD. (D) STAD.

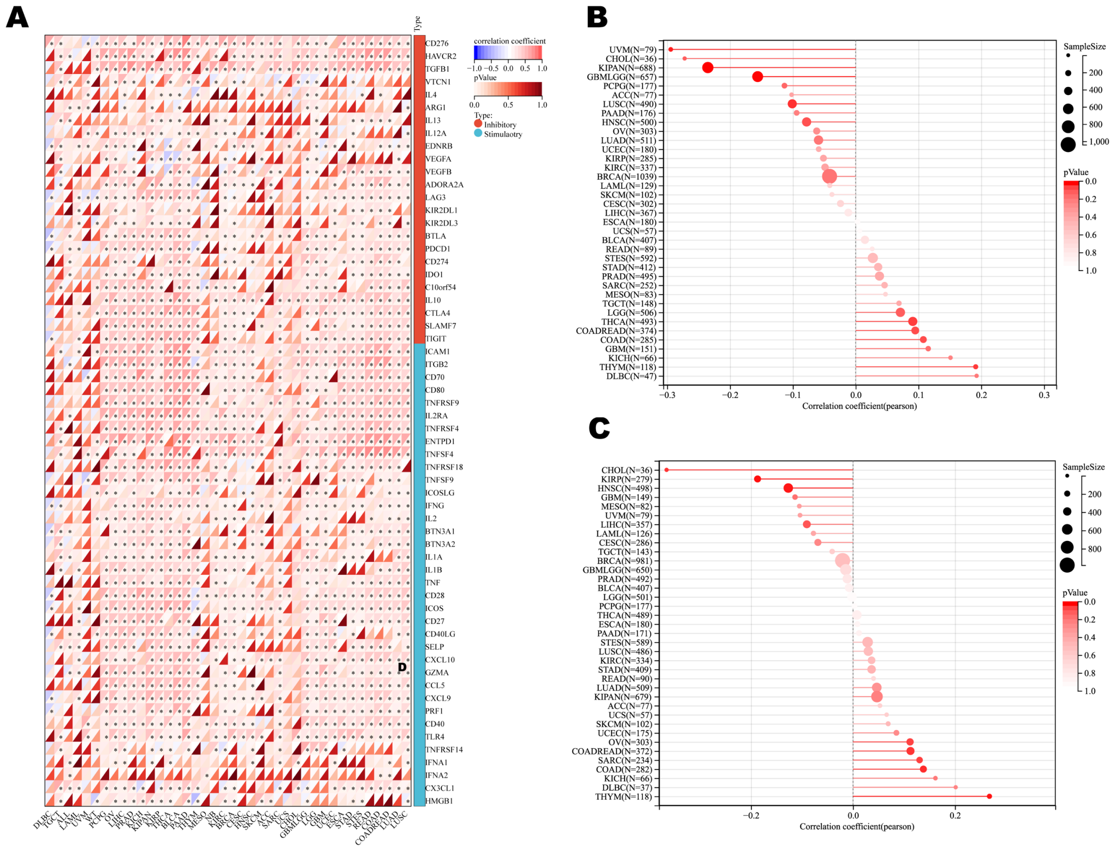

Supplementary Fig. 4 Correlation between FAP and immune check-points, MSI and TMB. (A) immune check-points. (B) MSI. (C) TMB.

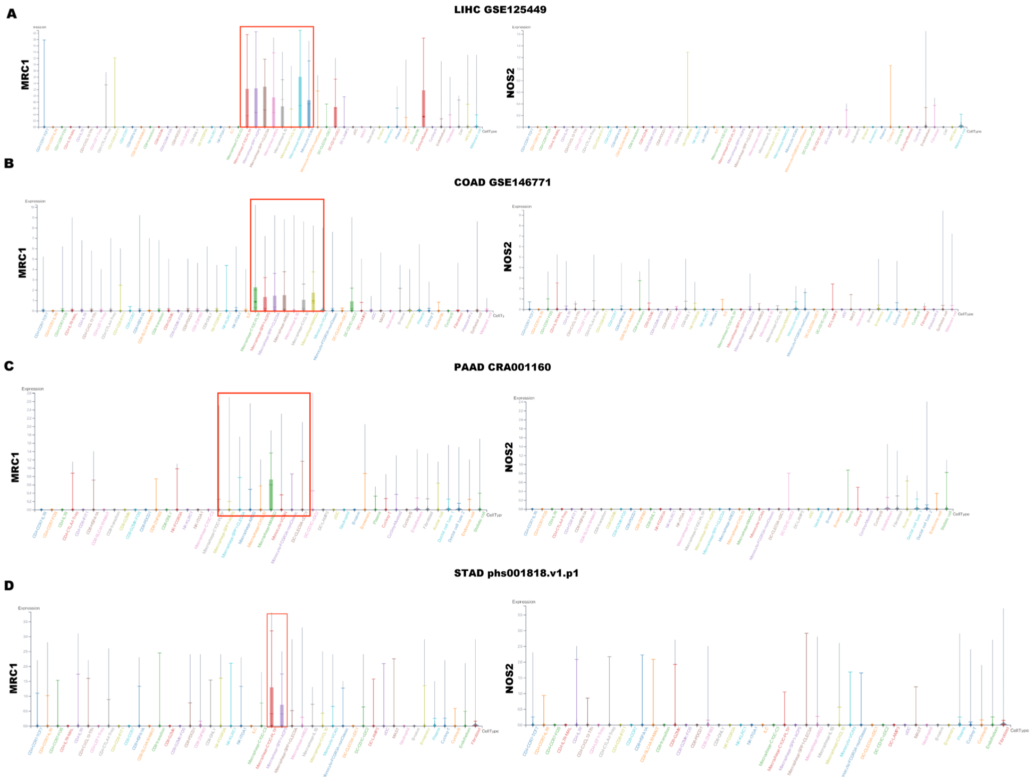

Supplementary Fig. 5 Single-cell sequencing analysis of NOS2 and MRC1 in gastrointestinal cancers. (E) LIHC. (F) COAD. (G) PAAD. (H) STAD.

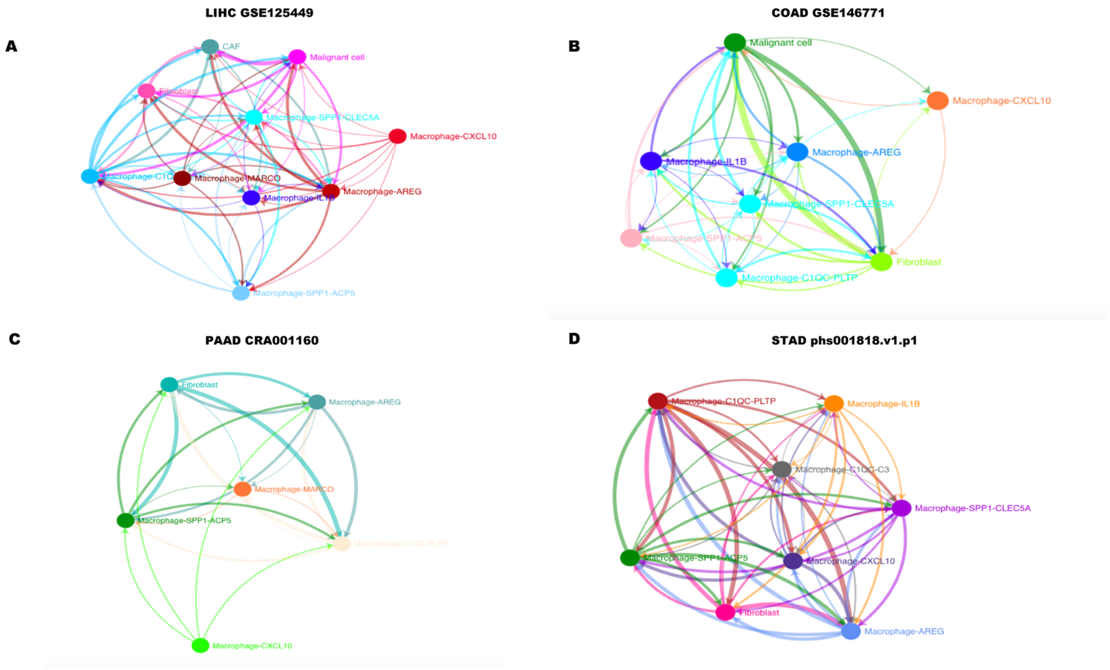

Supplementary Fig. 6 Single-cell sequencing analysis of fibroblasts and macrophages interactions in gastrointestinal cancers. (A) LIHC. (B) COAD. (C) PAAD. (D) STAD.
